# Predicting CDR status over 36 months with a recall‐based digital cognitive biomarker

**DOI:** 10.1002/alz.14213

**Published:** 2024-09-11

**Authors:** Davide Bruno, Ainara Jauregi‐Zinkunegi, Jason R. Bock, for the Alzheimer's Disease Neuroimaging Initiative

**Affiliations:** ^1^ School of Psychology Liverpool John Moores University Liverpool UK; ^2^ Embic Corporation Newport Beach California USA

**Keywords:** Alzheimer's Disease Assessment Scale–Cognitive subscale, Clinical Dementia Rating, cognitive assessment, digital cognitive biomarkers

## Abstract

**INTRODUCTION:**

Word‐list recall tests are routinely used for cognitive assessment, and process scoring may improve their accuracy. We examined whether Alzheimer's Disease Assessment Scale–Cognitive subscale (ADAS‐Cog) derived, process‐based digital cognitive biomarkers (DCBs) at baseline predicted Clinical Dementia Rating (CDR) longitudinally and compared them to standard metrics.

**METHODS:**

Analyses were performed with Alzheimer's Disease Neuroimaging Initiative (ADNI) data from 330 participants (mean age = 71.4 ± 7.2). We conducted regression analyses predicting CDR at 36 months, controlling for demographics and genetic risk, with ADAS‐Cog traditional scores and DCBs as predictors.

**RESULTS:**

The best predictor of CDR at 36 months was *M*, a DCB reflecting recall ability (area under the curve = 0.84), outperforming traditional scores. Diagnostic results suggest that *M* may be particularly useful to identify individuals who are unlikely to decline.

**DISCUSSION:**

These results suggest that *M* outperforms ADAS‐Cog traditional metrics and supports process scoring for word‐list recall tests. More research is needed to determine further applicability with other tests and populations.

**Highlights:**

- Process scoring and latent modeling were more effective than traditional scoring.
- Latent recall ability (*M*) was the best predictor of Clinical Dementia Rating decline at 36 months.
- The top digital cognitive biomarker model had odds ≈ 90 times greater than the top Alzheimer's Disease Assessment Scale–Cognitive subscale model.
- Particularly high negative predictive value supports literature on cognitive testing as a useful screen.
- Consideration of both cognitive and pathological outcomes is needed.

## 1 BACKGROUND

Word list recall tests are routinely used in clinical practice for the assessment of verbal memory ability, including in individuals with Alzheimer's disease (AD) and other dementias.^1^ Most commonly used neuropsychological tests of word‐list recall were developed for the purposes of identifying individuals with well‐defined memory loss predating dementia.^2^ However, the promise of disease‐modifying drugs for AD puts emphasis on identifying individuals who present *subtle* signs of underlying pathology at the earliest stages, as they may benefit the most from pharmacological interventions. Therefore, together with advancements in neuroimaging and fluid biomarkers, there is also a need for the development of more accurate neuropsychological testing, including with word lists, particularly as cognitive assessment typically is cost‐effective and requires relatively little training, compared to neuroimaging and fluid biomarkers capture.^3^ Process scoring and latent modeling of cognitive tests, which allow for the identification of underlying neurocognitive mechanisms of test performance, including word‐list recall tests, have shown potential to enhance test accuracy without requiring test redesign.^3^, ^4^, ^5^, ^6^, ^7^, ^8^, ^9^

One example is the use of hidden Markov modeling (HMM), a class of established cognitive models.^10^, ^11^ In the context of word‐list recall tests, an HMM characterizes episodic memory for a list item as existing in one of a set of latent storage states upon each observation of recall or non‐recall during the test. Retrieval parameters are associated with each storage state from which an item is capable of being recalled, and encoding parameters describe the transitions among the storage states.^12^ The sequence of encoding and retrieval transitions cannot be directly observed and is therefore “hidden” but quantifiable as probabilities of recall.

**FIGURE 1:**
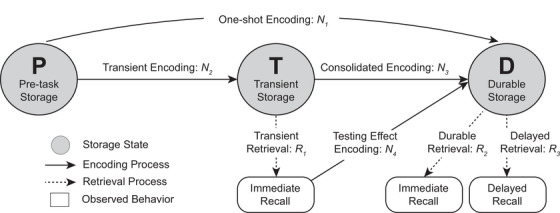
HBPC model. The model has three episodic memory storage states (*p*, *T*, and *D*), four processes of encoding into them (*N_1_*, *N_2_*, *N_3_*, and *N_4_*), and three processes of retrieval from them (*R_1_*, *R_2_*, and *R_3_*) that episodic memory of a word may exist in or transition through during each immediate and delayed free recall task. HBCP, hierarchical Bayesian cognitive processing; *p*, pre‐task storage; *T*, transient storage; *D*, durable storage; *N_1_*, one‐shot encoding; *N_2_*, transient encoding; *N_3_*, consolidated encoding; *N_4_*, testing effect encoding; *R_1_*, transient retrieval; *R_2_*, durable retrieval; *R_3_*, delayed retrieval.

A hierarchical Bayesian cognitive processing (HBCP) model is an HMM that has been applied to word‐list recall. The HBCP model posits that an item exists within one of three states upon each observation: pre‐task storage (*p*), which contains only semantic memory; transient storage (*T*), which contains temporarily stored episodic memory and enables immediate free recall (IFR); and durable storage (*D*), which contains episodic memory additionally available for delayed free recall (DFR) tasks. Three retrieval parameters quantify the probability of recall from the latter two states: Transient retrieval (*R_1_*), from *T* on IFR tasks; durable retrieval (*R_2_*), from *D* on IFR tasks; and delayed retrieval (*R_3_*), from *D* on DFR tasks. Four encoding parameters quantify the probability of an item transitioning from one state to another during each task: one‐shot encoding (*N_1_*), from *p* to *D*; transient encoding (*N_2_*), from *p* to *T*; consolidated encoding (*N_3_*), from *T* to *D* on a task subsequent to its encoding into *T*; and testing effect encoding (*N_4_*), from *T* to *D* upon successful recall from *T*. In the HBCP, these parameters are used as probabilities for each branch of a multinomial processing tree that reproduces the observed recall behavior (see Figure 1).^13^

Subsequent to generating the encoding and retrieval parameters, measures of recall ability can be estimated by recombining specific subsets of the multinomial processing tree branches comprising these parameters. Word recall through *T* (*M_1_*) includes the probabilities for *N_2_* and *R_1_*; word recall through *D* on IFR tasks (*M_2_*) includes *N_1_*, *N_2_*, *N_3_*, *N_4_*, and *R_2_*; and word recall through *D* on DFR tasks (*M_3_*) includes *N_1_*, *N_2_*, *N_3_*, *N_4_*, and *R_3_*. These memory (*M*) values represent generalized recall rates via various processes across words and tests. For example, an individual with an *M_3_* of 0.71 is expected to recall 7.1 words out of 10 on average across DFR tests. Collectively, the *N*, *R*, and *M* parameters are referred to as digital cognitive biomarkers (DCBs).

In this article, we test whether HBCP‐derived DCBs are useful predictors of cognitive decline, as measured by Clinical Dementia Rating (CDR). In particular, we aim to ascertain whether baseline estimates of *N*, *R*, and *M* yield more predictive power than the traditional Alzheimer's Disease Assessment Scale–Cognitive subscale (ADAS‐Cog) memory measures, such as immediate and delayed recall scores.

## 2 METHODS

### 2.1 Participants

Data were drawn from the Alzheimer's Disease Neuroimaging Initiative (ADNI) database, which has detailed methods^14^ reported elsewhere.^15^, ^16^ The ADNI is a longitudinal study launched in 2003 with measures of cognitive impairment and AD including clinical and neuropsychological assessment. To be included in this secondary data analysis study, participants had to have: ADAS‐Cog scores at baseline, from which traditional scores and DCBs were extracted; a CDR score at baseline and after a 36‐month follow‐up; and a polygenic hazard score (PHS) to determine genetic risk. The initial reference pool comprised 3418 participants, then reduced to 330 participants (mean age at baseline = 71.4, standard deviation [SD] = 7.2), after applying the inclusion criteria above. Of these, 184 were male and 146 were female. At baseline, 29 participants had a CDR score of 0, 300 scored 0.5, and 1 participant scored 1. At the 36‐month follow‐up visit, 64 participants had a CDR score of 0, 214 scored 0.5, 39 scored 1, 12 scored 2, and 1 participant scored 3. All activities for this study were approved by the ethics committees of the authors’ universities and completed in accordance with the Declaration of Helsinki. All participants provided informed consent before testing.

### 2.2 Materials

ADAS‐Cog^17^ is a neuropsychological test battery consisting of 13 subtests, including immediate and delayed word recall. In the word recall task, participants are visually and audibly presented with a list of 10 words and then asked to recall as many words as possible over three tasks: the order of presentation is varied across tasks, and the score is calculated as the mean number of words *not* recalled across the three tasks. After a 10‐minute delay with distraction, participants are once more asked to recall as many words as possible, this time without presentation, and the score is calculated as the number of words *not* recalled.

**RESEARCH IN CONTEXT**

1. **Systematic review**: The authors of the present study reviewed the literature (e.g., PubMed) on cognitive assessment scoring approaches and their ability to predict cognitive decline. Prior research indicates that process scoring and latent modeling approaches can improve cognitive test accuracy over traditional scoring.
2. **Interpretation**: Use of the *M* score, a digital cognitive biomarker derived from Alzheimer's Disease Assessment Scale–Cognitive subscale Word Recall test item responses with a hierarchical Bayesian cognitive processing model, enables more accurate prediction of cognitive decline at 36‐month follow‐up (model odds ≈ 90 times greater) than traditional immediate or delayed scores do. In particular, a greater negative predictive value indicates efficacy for ruling out patients not likely to decline.
3. **Future directions**: Cognitive testing should use process scoring or latent modeling to extract more information from participant responses. With enhanced information, these tests can be useful as screening tools to aid in clinical and research settings.

The CDR scale^18^ is a semi‐structured clinical interview that assesses six cognitive areas: memory, orientation, judgment and problem‐solving, community affairs, home and hobbies, and personal care. Each cognitive domain is then scored between 0 and 3, and to obtain a global score, the sum of each of the domains is calculated with equal weightings. The global CDR stages are 0, indicating normal cognition; 0.5, indicating mild cognitive impairment; and 1, 2, and 3, indicating mild, moderate, and severe dementia, respectively.

### 2.3 DCB

DCB scores were generated using ADAS‐Cog Word Recall item response data with the HBCP model, using Bayesian inference with a Markov chain Monte Carlo (MCMC) algorithm.^13^ Each assessment's observed sequence of recall and non‐recall was used to update prior information on DCB distributions for typical individuals in the general population who come from demographic groups (age, sex, and education level) specific to the participant who performed the assessment. The HBCP model additionally included adjustment for word presentation position effects on each of the three ADAS‐Cog English word lists. These DCBs are proprietary (Embic Corporation).

### 2.4 PHS

The Desikan AD PHS was computed based on a Cox proportional hazard regression model combining 31 AD‐associated single nucleotide polymorphisms (SNPs) with two apolipoprotein E variants (ε2/ε4), trained with genetic data from an independent cohort. The PHS, composed of a weighted score of 33 risk‐ or protection‐conferring SNPs, was calculated for each participant as previously described.^19^

### 2.5 Analysis plan

First, we carried out a longitudinal Bayesian linear regression analysis with CDR score at 36 months as outcome. Predictors were *N* (as average of *N_1_*, *N_2_*, *N_3_*, *N_4_*), *R* (as average of *R_1_*, *R_2_*, and *R_3_*), and *M* (as average of *M_1_*, *M_2_*, and *M_3_*), and the traditional ADAS‐Cog memory scores (immediate and delayed recall), all measured at baseline, and control variables were years of education, sex, PHS, age at baseline, and baseline CDR score. Credible intervals (CIs) were set to 95%. The prior was set to Jeffreys–Zellner–Siow, and the model prior was set to Uniform. One thousand MCMC simulations were conducted to determine parameters and compensate for possible violations of normality, but we also evaluated Q‐Q plots of residuals to estimate normality. After that, we carried out two sensitivity tests. We conducted a frequentist ordinal regression analysis with the same outcome and covariates but limited predictors to those that emerged from the initial Bayesian regression as strongest. Finally, to evaluate clinical validity of these predictive metrics, we conducted a frequentist logistic regression analysis: we used the increase in CDR score between baseline and 36 months of at least 0.5 as outcome and used the same predictor(s) and covariates (minus the baseline CDR score, already included in the change score) as in the ordinal analysis. Analyses were conducted using JASP (0.18.3)^20^ and SPSS (29.0; IBM).

## 3 RESULTS

Table 1 reports demographics, CDR, and memory scores in the cohort under examination.

**TABLE 1:** Means and SD for demographics, CDR score at baseline and 36‐month follow‐up, and memory scores, including DCBs *N*, *R*, and *M*.

	Mean	SD
Years of education	16.333	2.636
Age at baseline	71.397	7.247
PHS	0.339	0.779
Immediate recall	4.064	1.442
Delayed recall	4.748	2.569
*N*	0.431	0.054
*R*	0.593	0.048
*M*	0.710	0.083
CDR at baseline	0.458	0.145
CDR at 36 months	0.524	0.423

*Note*: *N*, *R*, and *M* are DCBs.

Abbreviations: CDR, Clinical Dementia Rating; DCB, digital cognitive biomarker; PHS,  polygenic hazard score; SD, standard deviation.

Results indicated that the CDR score at 36 months was best predicted by a model including only *M* (extreme evidence: BF_10_ > 1 billion, BF_inclusion_ = 26.9)—this model's odds were about three times as high (BF_M_ = 9.3 vs. BF_M_ = 3.2) as the next best model, including *M* and *R*. Compared to models including the traditional ADAS‐Cog immediate and delayed recall scores, the model with *M* alone performed > 3.5 times better than the model with immediate recall and *M* combined (BF_M _= 9.2 vs. BF_M _= 2.6, respectively), and > 5 times better than the model with delayed recall and *M* combined (whose BF_M_ was 1.7). The best model without DCBs (including both immediate and delayed recall) had a BF_M_ of 0.1: this finding indicates that the top model, with *M* alone, had model odds ≈ 90 times greater than the best model with only traditional ADAS‐Cog metrics.

The higher the *M* score, the lower the CDR score at 36 months (mean coefficient = –2.38, SD = 0.79): a cross‐sectional difference of 0.2 *M* points corresponds to a CDR difference of ≈ 0.5 (95% CIs: –4.52 to –1.05).

Given that the Q‐Q plot for the analysis above displayed some degree of non‐normality, we also carried out the same analysis on square‐root–transformed follow‐up CDR scores, which gave us more linear Q‐Q plots. The overall pattern of results was unchanged.

The sensitivity ordinal (frequentist) regression confirmed the overall findings above. The model fit was significant (χ^2^ [6] = 122.8, *p *< 0.001; Nagelkerke pseudo‐*R* ^2^ = 0.36), as was *M* (coefficient estimate = –12.92, standard error = 1.79, Wald coefficient = 52.32, *p* < 0.001).

**FIGURE 2:**
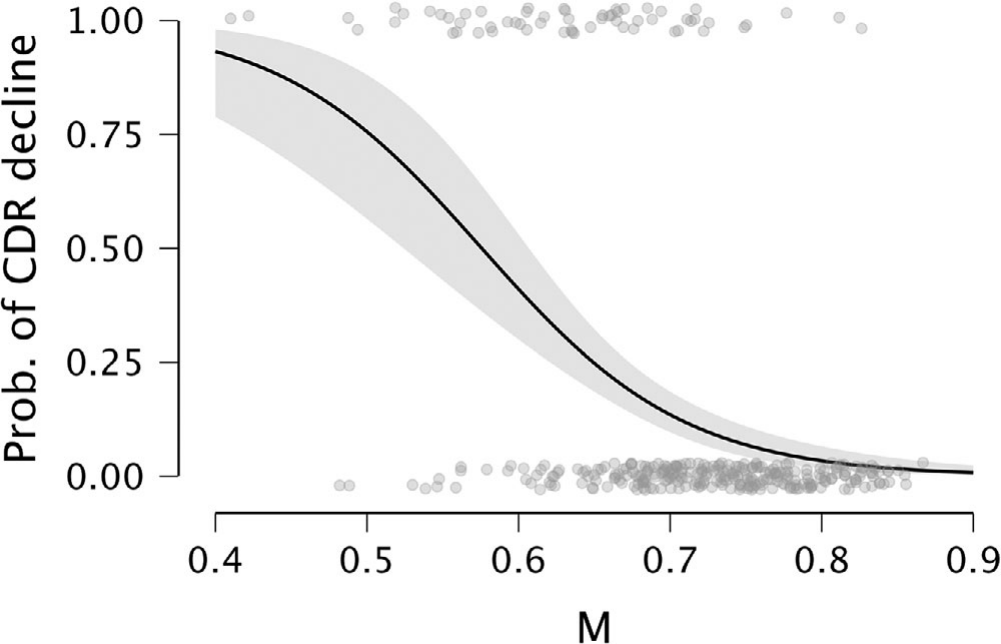
Conditional estimates plot with 95% confidence intervals (shaded area). The *y* axis represents the probability of CDR decline at follow‐up (36 months). The *x* axis represents *M* scores at baseline. Gray circles represent unique individuals’ data points. Circles at the bottom represent individuals who did not show CDR decline at follow‐up, whereas circles at the top represent CDR decliners at follow‐up. CDR, Clinical Dementia Rating.

**FIGURE 3:**
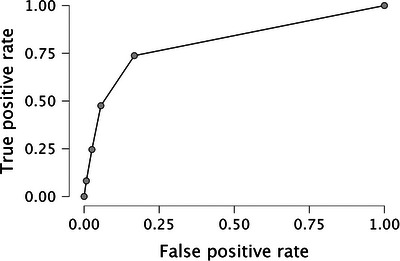
ROC plot comparing true positive rate (*y* axis) to false positive rate (*x* axis) in the frequentist logistic analysis. AUC = 0.84. ROC, receiver operating characteristic; AUC, area under the curve.

Finally, the frequentist logistic regression (269 individuals did not increase their CDR score by ≥ 0.5, and 61 did) showed that adding *M* to the model reduced the Akaike information criterion from 293.95 to 245.24. *M* was a significant predictor in this analysis (unstandardized coefficient estimate = –14.94, standardized coefficient estimate = –1.23, Wald coefficient = 39.75, odds ratio < 0.001, *p* < 0.001). Setting *M* at ≈ 0.58 yielded the following performance diagnostics: the area under the curve (AUC) was 0.84 (without *M* and only covariates the AUC was 0.71), the negative predictive value was 0.87 (258 correct rejections vs. 38 misses), and positive predictive value was 0.68 (23 hits vs. 11 false alarm). Furthermore, specificity was very high (0.96; 258 correct rejections vs. 11 false alarms), while sensitivity was lower (0.38; 23 hits vs. 38 misses). Figure 2 displays the association between the baseline *M* score and the probability of declining by at least 0.5 CDR points at follow‐up (36 months), and Figure 3 reports the receiver operating characteristic (ROC) curve.

## 4 DISCUSSION

In this analysis of ADNI data, we examined how HBCP‐derived DCBs (*N*, *R*, and *M*, indexing encoding, retrieval, and recall, respectively) compared to the traditional ADAS‐Cog assessment metrics (immediate and delayed recall scores) in predicting CDR score over a 36‐month span. Our analysis included 330 individuals and showed that DCB *M* was the better overall predictor in the test. These findings are in line with recent efforts demonstrating the validity of process metrics for early detection of cognitive impairment.^3^, ^4^, ^5^, ^6^, ^7^, ^21^, ^22^, ^23^, ^24^, ^25^

One observation is that the *M* metric, comparably to other cognitive tools in recent literature,^3^, ^25^, ^26^ appears more useful to exclude false negatives than to identify targets correctly, as indexed by the high negative predictive value and specificity. In other words, individuals scoring at *M* = 0.58 or higher at baseline were unlikely to decline after 36 months. While identification of positive cases appears more difficult with process scoring compared to, for example, fluid biomarkers,^27^, ^28^ a high negative predictive value still yields great utility. Typically, cognitive assessment is cost‐ and resource‐effective compared to most biomarker assessments, as cognitive assessments are cheaper (many are non‐proprietary), require less administrator training, and are less invasive for delivery. Therefore, especially in addressing global need where biomarker assessment is cost restrictive, there is value in cognitive screening which may help exclude individuals who, despite possible subjective concerns, are unlikely to be on a disease trajectory. Future assessments of *M* and related DCBs should include direct comparisons to state‐of‐the‐art fluid and imaging biomarkers.

Further research should also use the latest in process scoring and latent modeling. In the time since these analyses were performed, a second generation of DCBs was generated and included in the ADNI database as quantified cognitive processes (qCP). These qCPs account for additional differences in word features across the alternative lists of the ADAS‐Cog Word Recall test and include alternative *M* parameters representative of recall on specific immediate and delayed tasks. Future analyses can be performed to evaluate the predictive capability of these qCPs.

This secondary and preliminary assessment of HBCP‐derived DCBs has a definite limitation worth noting. The outcome (CDR score) is based upon clinical assessment of primarily cognitive function, and the predictors (*N*, *R*, *M*, and traditional immediate and delayed recall scores) are also measures of cognitive function, specifically memory, which risks issues of circularity. However, note: (1) the memory scores are not contributors to the CDR score, and (2) *M* outperformed other measures of recall performance. In addition to measures of cognitive function, further confirmation of the utility of HBCP‐derived DCBs should come from tests comparing this score to measures of pathology. Moreover, further studies should consider adding more demographic variety, such as including younger cohorts (as the present cohort was on average 70+ at baseline) and more ethnic diversity.

To conclude, word list memory tests are widely used for evaluating cognitive function, especially in AD research and screening. These tests vary in their elements, such as list length, number of learning attempts, sequence of presentation across attempts, and inclusion of semantic categories. Traditionally, scoring techniques, such as overall scores and more recently composite scoring, have not adequately addressed differences among these elements or their impact on learning and memory during the test.^29^ Recent advancements in process scoring and latent modeling offer promise in overcoming these limitations to provide better ways to assess cognitive performance. In this study, we show a specific example in HBCP‐derived DCBs, *M*.

## CONFLICT OF INTEREST STATEMENT

D.B. and A.J.Z. have no conflicts of interest. J.R.B. is an employee of Embic Corporation whose assessment outcomes were evaluated in this project. Author disclosures are available in the supporting information.

## CONSENT STATEMENT

ADNI data collection was approved by the ethics committees of the participating institutions, and completed in accordance with the Declaration of Helsinki. All participants provided informed consent before testing.

## Supporting information

Supporting information
